# The optimal target of vancomycin area under the curve in early or later phase on clinical outcomes and nephrotoxicity in patients with enterococcal infective endocarditis: how much is enough?

**DOI:** 10.1186/s12879-026-12993-5

**Published:** 2026-03-07

**Authors:** Aimatchara Worrasan, Manat Pongchaidecha, Wichai Santimaleeworagun

**Affiliations:** 1 Pharmacy Unit, Central Chest Institute of Thailand, Nonthaburi, 11000 Thailand; 2 Division of Pharmaceutical Care, Faculty of Pharmacy, Silpakorn University, Nakhon Pathom, 73000 Thailand; 3 Pharmaceutical Initiative for Resistant Bacteria and Infectious Diseases Working Group [PIRBIG], Faculty of Pharmacy, Silpakorn University, Nakhon Pathom, 73000 Thailand

**Keywords:** Vancomycin, AUC/MIC, Bayesian dosing, Nephrotoxicity, Enterococcal infective endocarditis

## Abstract

**Background:**

Enterococcal infective endocarditis (EIE) is associated with substantial morbidity and mortality, while optimal vancomycin pharmacodynamic targets for this condition remain uncertain.

**Objective:**

To determine the association between vancomycin area under the concentration–time curve to minimum inhibitory concentration ratio (AUC/MIC) and clinical outcomes in patients with EIE.

**Methods:**

This retrospective cohort study included adult patients with enterococcal infective endocarditis (EIE) who received intravenous vancomycin and had at least one measurable serum concentration. Vancomycin exposure parameters, including the 24-hour area under the concentration–time curve (AUC₀–₂₄, day 1) and steady-state AUC (AUCss), were retrospectively estimated using Bayesian modeling software and were not used to guide dose adjustment during patient care. The primary outcome was 30-day all-cause mortality, and secondary outcomes included microbiological failure and nephrotoxicity defined according to KDIGO criteria.

**Results:**

A total of 120 patients with EIE were included. The optimal cut-points for predicting 30-day survival were an AUC₀–₂₄/MIC ≥ 450 and an AUCss/MIC ≥ 420. Patients achieving these thresholds had significantly lower 30-day mortality compared with those below the thresholds. Adequate vancomycin exposure remained independently associated with improved survival. A prespecified AUCss ≥ 650 mg·h/L was associated with increased nephrotoxicity. In multivariate analysis, acute renal failure, septic shock, and low vancomycin AUC/MIC were independent predictors of 30-day mortality, whereas cardiac surgery demonstrated a protective effect.

**Conclusions:**

In patients with EIE, achieving AUC₀–₂₄/MIC ≥ 450 or AUCss/MIC ≥ 420 improves survival, whereas AUCss ≥ 650 mg·h/L significantly heightens nephrotoxicity risk. Early AUC-guided dose optimization and renal monitoring are crucial for balancing efficacy and safety in this high-risk population.

**Clinical trial registration:**

Not applicable.

**Supplementary Information:**

The online version contains supplementary material available at 10.1186/s12879-026-12993-5.

## Background / introduction

Infective endocarditis (IE) remains a severe infectious disease associated with high morbidity and mortality despite advances in antimicrobial therapy and cardiac surgery [1–3]. Its global incidence continues to rise due to aging populations, increasing comorbidities, and the widespread use of prosthetic valves and cardiac implantable electronic devices [2, 4–7]. Enterococcal infective endocarditis (EIE) accounts for approximately 13–18% of IE cases worldwide and 9–11% in Thailand [4, 8], and poses significant therapeutic challenges because of intrinsic antimicrobial resistance, biofilm formation, and limited options for synergistic therapy [9, 10].

Although *Enterococcus faecalis* and *Enterococcus faecium* are often grouped together in clinical studies, they differ substantially in antimicrobial susceptibility and treatment strategies. *E. faecalis* is generally susceptible to beta-lactam antibiotics and is preferentially treated with ampicillin-based regimens with or without synergistic gentamicin. In contrast, *E. faecium* frequently exhibits ampicillin resistance, making vancomycin or other non–beta-lactam agents necessary in many cases [9–11]. Consequently, vancomycin plays a central role in the treatment of EIE caused by *E. faecium*. However, vancomycin continues to play an important role in selected clinical scenarios, such as beta-lactam allergy or infections caused by beta-lactam–resistant enterococci, particularly in tertiary referral centers [11].

These clinical scenarios are particularly relevant in tertiary referral centers, where patients often present with severe disease, prior treatment exposure, or multiple contraindications to first-line therapy. Therefore, optimizing vancomycin exposure remains clinically important when vancomycin is selected as part of EIE management.

The pharmacodynamic efficacy of vancomycin is best described by the ratio of the 24-hour area under the concentration–time curve to the minimum inhibitory concentration (AUC/MIC) [12]. Current guidelines recommend AUC-guided dosing; however, these recommendations are largely extrapolated from evidence in methicillin-resistant *Staphylococcus aureus* bacteremia and endocarditis [13–15]. Whether these exposure targets are directly applicable to enterococcal endocarditis remains uncertain.

Emerging evidence from enterococcal bloodstream infections suggests that higher vancomycin AUC/MIC exposure is associated with improved clinical outcomes [16, 17]. A recent multicenter study from Thailand reported that achieving a steady-state vancomycin exposure (AUCss) of 420–650 mg·h/L was associated with improved survival and reduced nephrotoxicity in patients with enterococcal bloodstream infections [17]. However, that study did not specifically evaluate patients with EIE, a population characterized by higher bacterial burden, biofilm-associated infection, and prolonged antimicrobial therapy, leaving optimal pharmacodynamic targets in this setting unresolved [10, 17].

In addition, access to Bayesian software for AUC-guided dosing remains limited in many institutions, including several centers in Thailand, where vancomycin dosing often relies on conventional trough-based monitoring [12, 13]. Although Bayesian modeling allows early estimation of vancomycin exposure before steady state and may facilitate timely dose optimization in severe infections, clinical outcome data supporting this approach in EIE are scarce [18, 19].

Therefore, this study aimed to evaluate the association between vancomycin AUC/MIC exposure, including both early-phase and steady-state indices, and clinical outcomes such as 30-day mortality, microbiological failure, and nephrotoxicity in patients with EIE. The findings are intended to inform more effective and individualized vancomycin dosing strategies when vancomycin is clinically indicated in this high-risk population.

## Methods

### Study design and setting

This retrospective single-center cohort study was conducted at the Central Chest Institute of Thailand (CCIT), the national tertiary referral center for cardiovascular and pulmonary diseases located in Nonthaburi, under the Department of Medical Services, Ministry of Public Health. All adult patients diagnosed with enterococcal infective endocarditis (EIE) between January 2015 and October 2024 were screened for eligibility.

### Inclusion and exclusion criteria

Eligible participants were adults aged 18 years or older who were diagnosed with enterococcal infective endocarditis (EIE), classified as definite or possible according to the modified Duke criteria [20]. Patients were required to have at least one positive blood culture for *Enterococcus* spp., to have received intravenous vancomycin as part of their endocarditis treatment, and to have at least one evaluable serum vancomycin concentration available for pharmacokinetic analysis. Patients were excluded if they had received less than 48 h of intravenous vancomycin therapy or if their medical records were incomplete or they were lost to follow-up.

### Definitions

IE was defined according to the modified Duke criteria [20]. Peak vancomycin concentrations (Cpeak) were measured 1–2 h after completion of intravenous infusion, and trough concentrations (Ctrough) were obtained within 30 min prior to the next dose [12, 18].

### Antimicrobial susceptibility testing

Antimicrobial susceptibility testing was performed using the VITEK^®^ 2 automated system (bioMérieux, Marcy-l’Étoile, France) following the manufacturer’s instructions [21] and interpreted according to the CLSI M100 performance standards [22]. The reportable MIC range for vancomycin against *Enterococcus* spp. on this platform was 0.5–32 µg/mL.

### Vancomycin measurements

Serum vancomycin concentrations were measured using the Atellica^®^ Integrated Automation analyzer (Siemens Healthcare Diagnostics, Germany) employing a particle-enhanced turbidimetric inhibition immunoassay (PETINIA). The analytical quantification range was 3–50 µg/mL, extendable to 100 µg/mL with on-board dilution. The within-run and total coefficients of variation were both < 10% [23].

### AUC estimation

Vancomycin exposure was estimated retrospectively for research purposes using Bayesian modeling software (PrecisePK^®^ version 2.4.0; Healthware Inc., Los Angeles, CA, USA) [12, 18]. Patient-specific dosing histories, administration times, and measured serum vancomycin concentrations were entered into the program to estimate individualized pharmacokinetic parameters. These estimates were not available to treating clinicians and were not used to guide vancomycin dose adjustment during routine patient care.

The 24-hour area under the concentration–time curve (AUC₀–₂₄) was calculated using concentration data obtained within the first 24 h of therapy. Steady-state AUC (AUCss) was defined as the AUC estimated after at least five elimination half-lives. In patients who died or discontinued vancomycin therapy before reaching steady state, AUCss was extrapolated from the available concentration–time data using Bayesian modeling, which may introduce additional uncertainty in these estimates.

### Outcomes

The primary outcome of this study was 30-day all-cause mortality following the diagnosis of enterococcal IE. Secondary outcomes included 45-day mortality, in-hospital mortality, microbiological failure, and nephrotoxicity. Microbiological failure was defined as persistent *Enterococcus* spp. bacteremia beyond 7 days after initiation of vancomycin therapy. Nephrotoxicity was defined according to the KDIGO criteria [19], as an increase in serum creatinine ≥ 0.3 mg/dL within 48 h or ≥ 1.5 times the baseline value within 7 days.

### Statistical analysis

Statistical analyses were performed using SPSS version 27 (IBM Corp., Armonk, NY, USA). All tests were two-tailed, and a p-value < 0.05 was considered statistically significant. Baseline characteristics were summarized using descriptive statistics. Continuous variables were expressed as mean ± standard deviation (SD), median (interquartile range [IQR]), as appropriate. The normality of continuous variables was assessed using the Kolmogorov–Smirnov test. Continuous variables were compared using the independent-samples t-test or the Mann–Whitney U test, as appropriate, while categorical variables were compared using the Chi-square test or Fisher’s exact test.

Univariable logistic regression analyses were performed to identify factors associated with 30-day all-cause mortality and nephrotoxicity. Variables with a p-value < 0.10 in univariable analysis, as well as variables considered clinically relevant based on prior literature, were included in multivariable logistic regression models. Results were reported as adjusted odds ratios (aORs) with 95% confidence intervals (CIs). Model calibration was assessed using the Hosmer–Lemeshow goodness-of-fit test, and multicollinearity was evaluated using variance inflation factors (VIFs).

Receiver operating characteristic (ROC) curve analysis was conducted to evaluate the ability of vancomycin exposure indices (AUC₀–₂₄/MIC and AUCss/MIC) to predict 30-day mortality. Optimal cutoff values were determined using the Youden Index, and the corresponding area under the ROC curve (AUROC) with 95% confidence intervals was reported.

For nephrotoxicity analysis, the association between vancomycin exposure (AUCss) and nephrotoxicity was evaluated using logistic regression, with AUCss analyzed as a categorical variable using a threshold of ≥ 650 mg·h/L.

## Results

### Baseline characteristics

A total of 120 patients met the inclusion criteria. The median age was 71 (IQR 68–82) years, and 67.5% were male. The median Charlson comorbidity index was 5 (IQR 3–7), with chronic heart failure (41.7%), diabetes mellitus (27.5%), and chronic kidney disease (15.8%) being the most prevalent comorbidities.

Native-valve infective endocarditis was present in 57.5% of patients, followed by prosthetic-valve endocarditis in 40.0% and cardiac implantable electronic device–related endocarditis in 2.5%. Aortic valve involvement was most common (66.7%), with mitral (27.5%) and tricuspid (3.3%) valve involvement observed less frequently. The median left ventricular ejection fraction was 50% (IQR 30–65), and the median vegetation size was 8 mm (IQR 3–14). Patient characteristics are summarized in Table 1.

**Table 1:** Patient characteristics of enterococcal infective endocarditis (*n* = 120)

Demographics	Value
Age, years – median (IQR)	71 (68–82)
Male sex – n (%)	81 (67.5)
Comorbidities – n (%)	
• Diabetes mellitus	33 (27.5)
• Chronic heart failure	50 (41.7)
• Chronic lung disease	22 (18.3)
• Cerebrovascular disease	9 (7.5)
• Chronic kidney disease	19 (15.8)
• Chronic liver disease	5 (4.2)
• Autoimmune disease	1 (0.8)
• Charlson comorbidity index – median (IQR)	5.0 (3–7)
Infection characteristics	
• Native-valve IE – n (%)	69 (57.5)
• Prosthetic-valve IE – n (%)	48 (40.0)
• CIED-related IE – n (%)	3 (2.5)
Valve involvement – n (%)	
• Aortic	80 (66.7)
• Mitral	33 (27.5)
• Tricuspid	4 (3.3)
• Device lead involvement (CIED)	3 (2.5)
Diagnostic classification	
• Definite IE – n (%)	99 (82.5)
• Possible IE – n (%)	21 (17.5)
Cardiac function and vegetations	
• Ejection fraction (%) – median (IQR)	50 (30–65)
• Vegetation size (mm) – median (IQR)	8 (3–14)
Clinical complications – n (%)	
• New-onset/worsening heart failure	56 (46.7)
• Persistent bacteremia	30 (25.0)
• Septic shock	13 (10.8)
• CNS emboli	14 (11.7)
• Pulmonary emboli	3 (2.5)
• Acute renal failure	37 (30.8)
• Renal abscess	1 (0.8)
• Osteomyelitis	4 (3.3)
• Splenic abscess	10 (8.3)
• Conduction abnormality	8 (6.7)
Severity	
• Critically ill – n (%)	86 (71.7)
• APACHE II score – median (IQR)	18 (16–21)
Microbiology	
• *E*. *faecalis* – n (%)	38 (31.7)
• *E*. *faecium* – n (%)	80 (66.7)
• Other Enterococci – n (%)	2 (1.7)
Vancomycin MIC – n (%)	
• MIC ≤ 0.5 µg/mL	20 (16.7)
• MIC = 1 µg/mL	97 (80.8)
• MIC = 2 µg/mL	3 (2.5)
High-level gentamicin resistance	13 (10.8)
Treatment	
• Cardiac surgery – n (%)	84 (70.0)
• Duration of vancomycin therapy, days – median (IQR)	42 (41–48)
• Duration of gentamicin therapy, days – median (IQR)	14 (11–14)
Concomitant nephrotoxic drugs – n (%)	
• Gentamicin	85 (70.8)
• Furosemide	46 (38.3)
• Colistin	7 (5.8)
• Piperacillin/tazobactam	5 (4.2)

Abbreviations: CIED, cardiac implantable electronic device; APACHE II, Acute Physiology and Chronic Health Evaluation II; MIC, minimum inhibitory concentration

Among the 38 patients with *Enterococcus faecalis* infective endocarditis, the majority of isolates were susceptible to ampicillin, with no isolate exhibiting an ampicillin MIC ≥ 16 µg/mL. Vancomycin was used primarily due to clinical constraints rather than antimicrobial resistance, most commonly documented penicillin or β-lactam allergy (*n* = 22). Additional reasons included prior treatment with ampicillin plus gentamicin followed by a switch to vancomycin due to clinical considerations (*n* = 7), continuation of empiric vancomycin without de-escalation (*n* = 3), adverse drug reaction (*n* = 1), and referral from other institutions without a clearly documented rationale for antibiotic selection (*n* = 1).

### Pharmacokinetic and pharmacodynamic parameters

Pharmacokinetic and pharmacodynamic parameters are summarized in Table 2. The mean body weight and BMI of the cohort were 61.5 ± 10.3 kg and 22.0 ± 1.7 kg/m², respectively. The mean loading dose of vancomycin was 23.4 ± 4.4 mg/kg, and the total dose administered during the first 24 h averaged 39.4 ± 4.8 mg/kg.

The mean elimination half-life was 15.2 ± 11.5 h. The mean AUC₀–₂₄ and steady-state AUC (AUCss) were 508.6 ± 94.9 mg·h/L and 515.2 ± 90.9 mg·h/L, respectively. The corresponding mean AUC/MIC ratios were 592.0 ± 247.7 and 598.4 ± 243.6, respectively.

**Table 2:** Pharmacokinetic and pharmacodynamic parameters of vancomycin in patients with enterococcal infective endocarditis (*n* = 120)

Parameter	Value
Weight, kg – mean ± SD	61.53 ± 10.25
Height, cm – mean ± SD	166.23 ± 9.75
BMI, kg/m² – mean ± SD	22.02 ± 1.66
Serum creatinine, mg/dL – median (IQR)	1.01 (0.74–1.17)
Dosing regimen	
• Loading dose, mg/kg – mean ± SD	23.41 ± 4.42
• Total dose in first 24 h, mg/kg – mean ± SD	39.38 ± 4.81
Pharmacokinetic parameters	
• Elimination rate constant (Ke), h⁻¹ – mean ± SD	0.057 ± 0.02
• Half-life, h – mean ± SD	15.16 ± 11.50
• Volume of distribution, L/kg – mean ± SD	0.84 ± 0.06
• Clearance, L/h – mean ± SD	2.73 ± 0.91
Exposure parameters	
• AUC₀–₂₄, mg·h/L – mean ± SD	508.56 ± 94.97
• AUCss, mg·h/L – mean ± SD	515.16 ± 90.88
• AUC₀–₂₄/MIC – mean ± SD	591.97 ± 247.71
• AUCss/MIC – mean ± SD	598.43 ± 243.61

Abbreviations: AUC₀–₂₄, area under the concentration–time curve during the first 24 h; AUCss, steady-state AUC; Ke, first-order elimination rate constant; MIC, minimum inhibitory concentration; BMI, body mass index; IQR, interquartile range

### Clinical outcomes

Clinical outcomes are summarized in Table 3. Thirty-day, 45-day, and in-hospital mortality rates were 15.8%, 20.8%, and 22.5%, respectively. Microbiological failure occurred in 20.8% of patients, and nephrotoxicity in 28.3%. The median length of hospital stay was 42 days (IQR 41–48).

**Table 3:** Clinical outcomes of patients with enterococcal infective endocarditis treated with vancomycin (*n* = 120)

Clinical outcome	Value
30-day mortality – n (%)	19 (15.8)
45-day mortality – n (%)	25 (20.8)
In-hospital mortality – n (%)	27 (22.5)
Microbiological failure – n (%)	25 (20.8)
Nephrotoxicity – n (%)	34 (28.3)
Length of hospital stay, days – median (IQR)	42 (41–48)

Definitions of outcomes are provided in Methods

### Association between vancomycin exposure and clinical outcomes

**Fig. 1:**
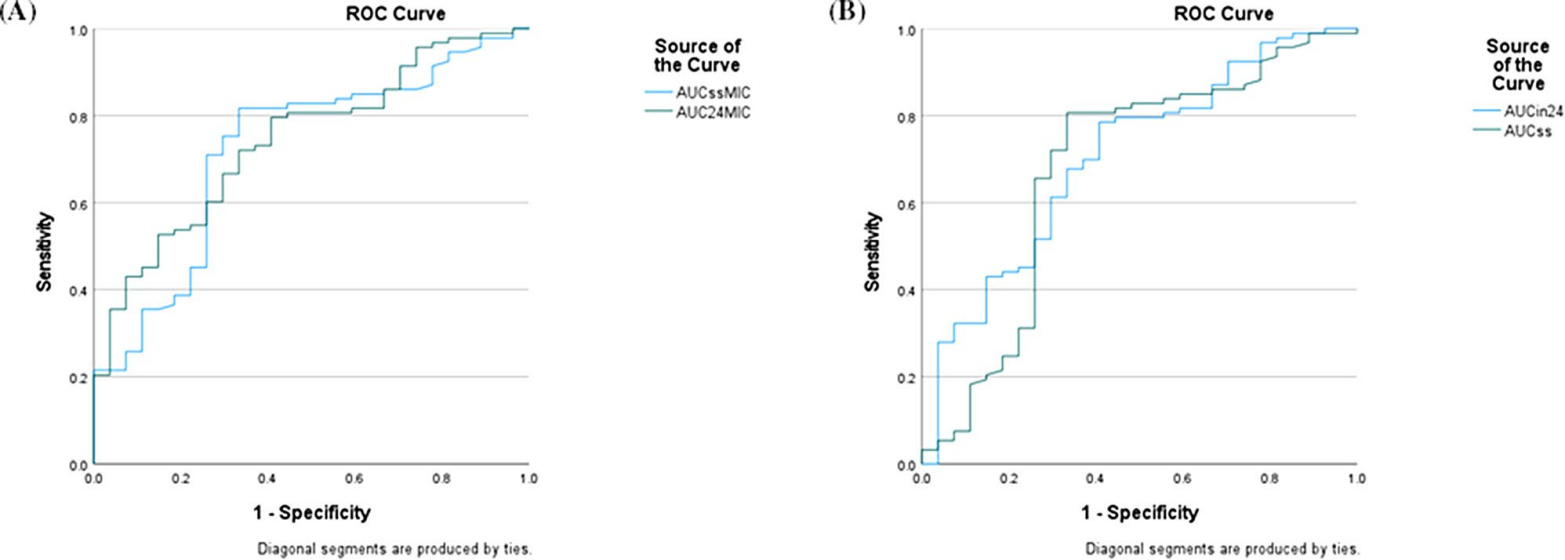
Receiver operating characteristic (ROC) curves of vancomycin exposure parameters for predicting 30-day mortality in patients with enterococcal infective endocarditis. (**A**) ROC curves of AUC₀–₂₄/MIC and AUCss/MIC. (**B**) ROC curves of AUC₀–₂₄ and AUCss

The association between vancomycin exposure indices and clinical outcomes was examined. Receiver operating characteristic (ROC) analysis was performed to evaluate the ability of vancomycin exposure indices to predict 30-day all-cause mortality, with optimal cutoff values of 450.1 for AUC₀–₂₄/MIC and 419.7 for AUCss/MIC, corresponding sensitivities of 80.2% and 91.1% and specificities of 63.2% and 57.9%, respectively (Fig. 1).

Patients achieving an AUC₀–₂₄/MIC ≥ 450 had lower 30-day mortality compared with those below the threshold (10.3% vs. 26.2%, *p* = 0.046), with similar reductions observed for 45-day mortality, in-hospital mortality, and microbiological failure; consistent findings were observed for an AUCss/MIC ≥ 420, while nephrotoxicity did not differ significantly between exposure groups (all *p* > 0.05) (Table 4).

**Table 4:** Outcome of vancomycin AUC/MIC by Bayesian in patients with enterococcal infective endocarditis (*n* = 120)

Outcomes *n* (%)	AUC₀–₂₄/MIC		*p*-value	AUCss/MIC		*p*-value
	< 450 (*n* = 42)	≥ 450 (*n* = 78)		< 420 (*n* = 28)	≥ 420 (*n* = 92)	
30-day mortality	11 (26.19)	8 (10.26)	0.046	9 (32.14)	10 (10.87)	0.003
45-day mortality	14 (33.33)	11 (14.10)	0.001	11 (39.29)	14 (15.22)	0.023
In-hospital mortality	14 (33.33)	13 (16.67)	0.003	12 (42.86)	15 (16.30)	0.014
Microbiological failure	11 (26.19)	14 (17.95)	0.036	12 (42.86)	13 (14.13)	0.008
Nephrotoxicity	9 (21.43)	25 (32.05)	0.246	7 (25.00)	27 (29.35)	0.658

**Fig. 2:**
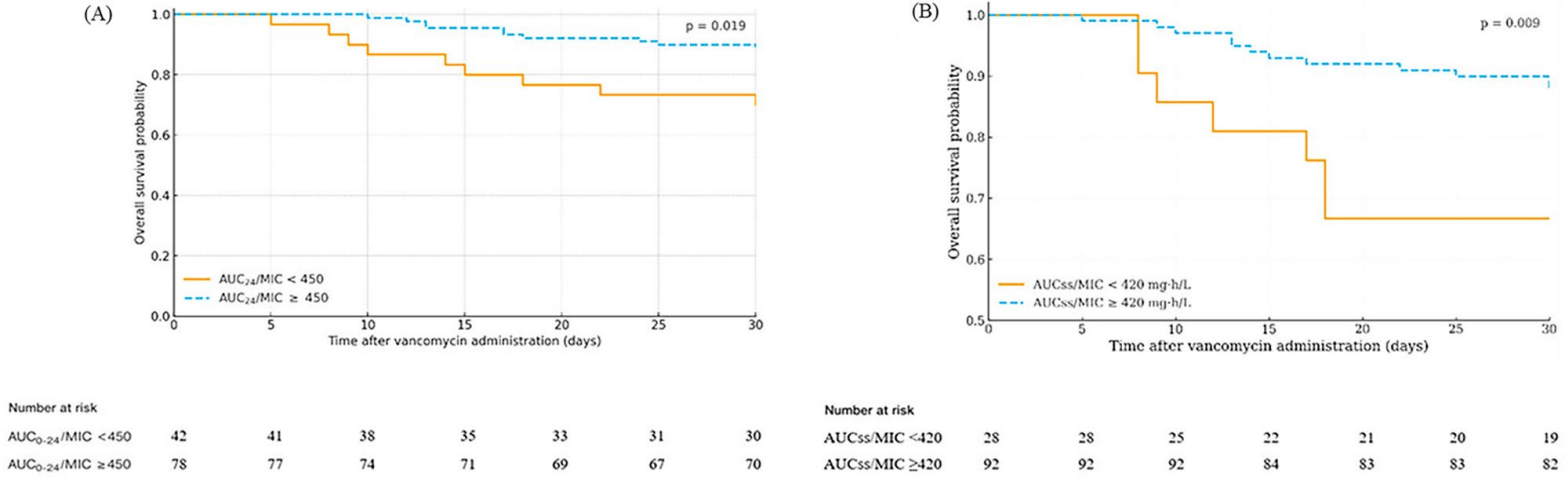
Kaplan–Meier survival curves for patients with enterococcal infective endocarditis treated with vancomycin. (**A**) AUC₀–₂₄/MIC < 450 vs. ≥450 (log-rank *p* = 0.019). (**B**) AUCss/MIC < 420 vs. ≥420 (log-rank *p* = 0.009)

Kaplan–Meier survival analysis showed improved 30-day survival among patients with AUC₀–₂₄/MIC ≥ 450 compared with those < 450 (log-rank *p* = 0.019); similar findings were observed for AUCss/MIC ≥ 420 versus < 420 (log-rank *p* = 0.009) (Fig. 2).

### Numbers at risk are shown below each curve

**Fig. 3:**
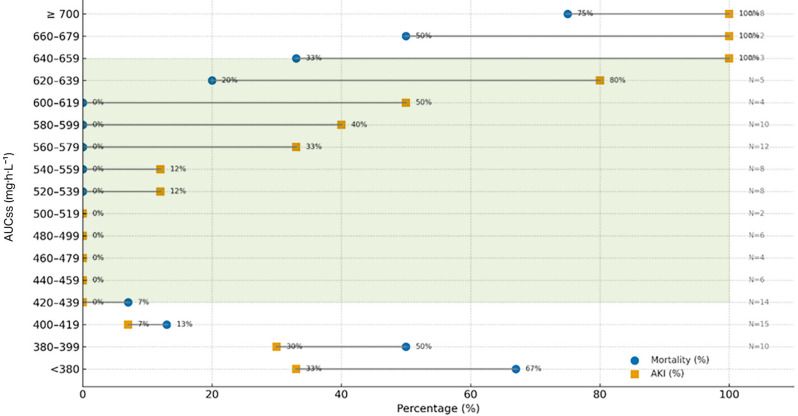
Relationship between steady-state vancomycin exposure (AUCss, mg·h/L), 30-day mortality, and nephrotoxicity in patients with enterococcal infective endocarditis

An exposure–response pattern was observed, with lower 30-day mortality at increasing steady-state vancomycin exposure, while the incidence of nephrotoxicity increased at higher AUCss values, particularly above 650 mg·h/L (Fig. 3).

### Factor associated with 30-day mortality

Univariate analysis identified several factors associated with increased 30-day mortality, including prosthetic IE, vegetation size ≥ 10 mm, new-onset heart failure, acute renal failure, septic shock, absence of cardiac surgery, and low vancomycin exposure (AUCss/MIC < 420).

In the multivariate model, new-onset heart failure (aOR 4.96; *p* = 0.040), acute renal failure (aOR 3.92; *p* = 0.042), and septic shock (aOR 4.85; *p* = 0.045) remained independent predictors of 30-day mortality. Cardiac surgery (aOR 0.20; *p* = 0.013) and adequate vancomycin exposure (AUCss/MIC ≥ 420) (aOR 0.14; *p* = 0.011) were significantly protective (Table 5).

**Table 5:** Univariate and multivariate logistic regression analyses of factors associated with 30-day mortality

Characteristic	Univariate analysis				Multivariate analysis		
	OR	95% CI	*p*-value		aOR	95% CI	*p*-value
Prosthetic IE	2.84	1.30–7.84	0.044		2.79	0.69–11.22	0.149
Vegetation ≥ 10 mm	4.17	1.44–12.1	0.008		3.16	0.54–18.30	0.200
New-onset heart failure	2.92	1.03–8.31	0.044		4.96	1.07–22.97	0.040
Persistent bacteremia	2.61	0.91–7.28	0.067		2.55	0.66–9.86	0.175
Acute renal failure	3.04	1.12–8.29	0.029		3.92	1.05–14.57	0.042
Septic shock	4.15	1.19–14.50	0.026		4.85	1.03–22.72	0.045
Cardiac surgery	0.31	0.11–0.85	0.023		0.20	0.05–0.71	0.013
AUCss/MIC ≥ 420	0.28	0.09–0.82	0.020		0.14	0.03–0.61	0.011

Abbreviations: OR, odds ratio; aOR, adjusted odds ratio; CI, confidence interval; MIC, minimum inhibitory concentration

### Predictor of nephrotoxicity

In univariate analysis, age ≥ 70 years, reduced renal function (CrCl < 60 mL/min), concomitant nephrotoxic drugs, septic shock, and high vancomycin exposure (AUCss ≥ 650 mg·h/L) were significantly associated with increased nephrotoxicity.

In the multivariate model, age ≥ 70 years (aOR 4.75; *p* = 0.009), CrCl < 60 mL/min (aOR 2.63; *p* = 0.045), concomitant nephrotoxic medications (aOR 3.24; *p* = 0.045), and AUCss ≥ 650 mg·h/L (aOR 5.81; *p* = 0.027) remained independent predictors of nephrotoxicity. Septic shock was no longer associated with nephrotoxicity after adjustment (*p* = 0.604). Full model results are shown in Table 6.

**Table 6:** Univariate and multivariate logistic regression analyses of factors associated with nephrotoxicity

Characteristic	Univariate analysis				Multivariate analysis		
	OR	95% CI	*p*-value		aOR	95% CI	*p*-value
Age ≥ 70 yrs	4.18	1.47–11.83	0.007		4.75	1.48–15.21	0.009
CrCl < 60 mL/min	3.05	1.28–7.29	0.012		2.63	1.02–6.75	0.045
Concomitant nephrotoxic drugs	4.01	1.29–12.48	0.016		3.24	1.03–10.22	0.045
Septic shock	3.45	1.07–11.10	0.038		1.50	0.33–6.88	0.604
AUCss ≥ 650 mg·h/L	4.39	1.15–16.71	0.022		5.81	1.22–27.62	0.027

Abbreviations: OR, odds ratio; aOR, adjusted odds ratio; CI, confidence interval; CrCl, creatinine clearance; AUCss, steady-state area under the concentration–time curve

## Discussion

This study demonstrates that vancomycin exposure, assessed using both early-phase and steady-state AUC/MIC indices, is strongly associated with mortality outcomes in patients with enterococcal infective endocarditis (EIE). Higher vancomycin exposure, particularly achieving AUC₀–₂₄/MIC ≥ 450 and AUCss/MIC ≥ 420, was associated with lower 30-day mortality, 45-day mortality, and in-hospital mortality. These findings suggest that adequate vancomycin exposure is clinically relevant in EIE when vancomycin is used and support the concept that both early target attainment and sustained exposure contribute to improved survival.

The predominance of *Enterococcus faecium* in our cohort reflects real-world practice in tertiary referral centers, where patients with more severe disease, prior treatment failure, or antimicrobial resistance are frequently encountered. Because *E. faecium* commonly exhibits ampicillin resistance and reduced aminoglycoside synergy, vancomycin remains a cornerstone therapy in this population. Importantly, vancomycin was also used in selected cases of ampicillin-susceptible *Enterococcus faecalis* due to beta-lactam allergy, intolerance, renal dysfunction limiting aminoglycoside use, or inadequate clinical response to guideline-recommended beta-lactam regimens prior to referral. These clinical scenarios underscore the ongoing relevance of vancomycin in EIE management and highlight the importance of optimizing vancomycin exposure when it is clinically indicated.

Our findings are consistent with established pharmacokinetic/pharmacodynamic principles derived from serious Gram-positive infections, particularly methicillin-resistant *Staphylococcus aureus* (MRSA) bacteremia and endocarditis, where an AUC/MIC threshold of approximately 400 has been associated with improved clinical outcomes [12, 24–26]. In addition, previous studies, such as the work by Lodise et al. [27], have shown that optimizing vancomycin exposure using AUC-guided dosing strategies in MRSA bloodstream infections can lead to improved clinical outcomes. The 2020 IDSA/ASHP consensus guidelines recommend an AUC/MIC target range of 400–600 to balance efficacy and nephrotoxicity [12, 28]. Although these recommendations are largely extrapolated from MRSA data, our results suggest that similar exposure targets may be applicable to EIE when vancomycin is used. Notably, the steady-state exposure range identified in our cohort (AUCss/MIC ≥ 420) closely aligns with the therapeutic window reported in a recent multicenter Thai study of enterococcal bloodstream infections, which demonstrated improved survival and reduced nephrotoxicity within an AUCss range of 420–650 [17]. In addition, our study extends this evidence by demonstrating that early vancomycin exposure is also clinically relevant, as achieving AUC₀–₂₄/MIC ≥ 450 was associated with improved survival outcomes. Together, these findings suggest that both early and sustained vancomycin exposure within an appropriate range may be important determinants of outcome in EIE.

The importance of early-phase vancomycin exposure is biologically plausible in the context of EIE, a condition characterized by high bacterial burden, and a risk of rapid clinical deterioration [2, 11]. Bayesian-guided dosing allows estimation of AUC prior to steady state and may facilitate timely dose optimization during the critical early phase of therapy [9, 12]. Early attainment of therapeutic exposure may enhance bacterial killing before steady state is reached, particularly in critically ill patients or those requiring surgical intervention.

With regard to safety, no clear independent association was observed between higher vancomycin AUC/MIC exposure and nephrotoxicity in this cohort. Instead, nephrotoxicity appeared to be more strongly associated with patient-related factors, including older age, baseline renal dysfunction, and concomitant nephrotoxic drug exposure. These findings are consistent with previous reports suggesting that patient characteristics and comorbidities play a major role in vancomycin-associated kidney injury [12, 24, 29]. In our study, nephrotoxicity was associated with higher steady-state vancomycin exposure, particularly at AUCss values ≥ 650 mg·h/L. This threshold was supported by the observed exposure–toxicity relationship in our cohort and is consistent with ranges reported in prior studies.

Overall, this study highlights the potential clinical value of AUC-guided vancomycin dosing in EIE when vancomycin is clinically indicated. Our findings suggest that achieving adequate vancomycin exposure early in therapy and maintaining appropriate steady-state exposure may improve survival outcomes without substantially increasing nephrotoxicity risk. Nevertheless, prospective multicenter studies are needed to validate these exposure targets and to further refine individualized dosing strategies for this high-risk population.

### Limitations

This study has several important limitations. First, its retrospective single-center design introduces referral bias, as our institution functions as a cardiothoracic tertiary center where more severe and antimicrobial-resistant EIE cases, particularly those due to *E. faecium*, are commonly managed.

Second, Some patients with enterococcal infective endocarditis (EIE) were ampicillin-susceptible, and while ampicillin, as the first-line therapy, may have yielded better outcomes [30, 31], vancomycin was used due to penicillin/β-lactam allergy, initial ampicillin plus gentamicin failure, or other clinical factors. Due to the small sample size (38 patients, or 31.7% of the cohort), subgroup analysis was not performed as it would not yield statistically reliable results.

Third, antimicrobial susceptibility data were limited to routine testing, and vancomycin MICs were obtained using automated systems rather than broth microdilution, which may introduce inter-assay variability affecting AUC/MIC interpretation [32–34].

Fourth, the wide variability in vancomycin treatment duration likely reflects early mortality, therapy modification, or surgical referral, but these factors could not be fully separated in this dataset. In addition, a small number of patients died or discontinued vancomycin before reaching pharmacokinetic steady state; in these cases, AUCss was extrapolated from limited concentration–time data using Bayesian modeling, which may have introduced additional uncertainty. However, this accounted for only 2.5% of the cohort and is unlikely to have materially affected the overall findings.

Fifth, the number of nephrotoxicity events was relatively small, limiting precision in exposure–toxicity analysis. Finally, Bayesian AUC estimation was based on trough-only sampling in some patients, which may introduce variability compared with rich sampling, although prior studies support its clinical reliability. Prospective multicenter studies using standardized MIC testing and predefined AUC-guided dosing algorithms are needed to confirm and refine these exposure thresholds.

## Conclusions

In this retrospective cohort study of patients with enterococcal infective endocarditis treated with vancomycin, higher vancomycin exposure assessed using early-phase and steady-state AUC/MIC indices was associated with lower mortality. Achieving AUC₀–₂₄/MIC ≥ 450 and AUCss/MIC ≥ 420 was consistently associated with improved 30-day, 45-day, and in-hospital survival. Additionally, the higher absolute vancomycin exposure was associated with an increased risk of nephrotoxicity. Overall, these findings suggest that early attainment and maintenance of adequate vancomycin exposure using AUC-based monitoring and individualized dosing may be important for optimizing outcomes in patients with enterococcal infective endocarditis when vancomycin is clinically indicated.

## Supplementary Information

Below is the link to the electronic supplementary material.

Supplementary Material 1

Supplementary Material 2

## Data Availability

The datasets generated and/or analyzed during the current study are available from the corresponding author on reasonable request.

## Abbreviations

AKI: Acute kidney injury
APACHE II: Acute Physiology and Chronic Health Evaluation II
AST: Antimicrobial susceptibility testing
AUC: Area under the concentration–time curve
AUC₀–₂₄: Area under the concentration–time curve during the first 24 h
AUC/MIC: Area under the concentration–time curve to minimum inhibitory concentration ratio
AUCss: Steady-state area under the concentration–time curve
CI: Confidence interval
CIED: Cardiac implantable electronic device
CLSI: Clinical & Laboratory Standards Institute
CrCl: Creatinine clearance
Cpeak: Peak (maximum) serum concentration
Ctrough: Trough (minimum) serum concentration
EIE: Enterococcal infective endocarditis
IE: Infective endocarditis
IQR: Interquartile range
KDIGO: Kidney Disease: Improving Global Outcomes
MIC: Minimum inhibitory concentration
MRSA: Methicillin-resistant *Staphylococcus aureus*
OR: Odds ratio
aOR: Adjusted odds ratio
PD: Pharmacodynamics
PETINIA: Particle-enhanced turbidimetric inhibition immunoassay
PK: Pharmacokinetics
PK/PD: Pharmacokinetic/pharmacodynamic
REC: Research Ethics Committee
ROC: Receiver operating characteristic
SD: Standard deviation
TDM: Therapeutic drug monitoring
