# Supplementary material for: The optimal target of vancomycin area under the curve in early or later phase on clinical outcomes and nephrotoxicity in patients with enterococcal infective endocarditis: how much is enough?
Source: BMC Infect Dis. 2026 Mar 7;26:759. doi: 10.1186/s12879-026-12993-5 (PMC13081359; doi:10.1186/s12879-026-12993-5)
Supplement: Supplementary file 1 — Supplementary Material 1 [file 12879_2026_12993_MOESM1_ESM.docx]

**Supplementary Table 1. Baseline characteristics stratified by early vancomycin exposure (AUC₀–₂₄/MIC <450 vs ≥450)**

| **Variable** | **AUC₀–₂₄/MIC <450 (n=42)** | **AUC₀–₂₄/MIC ≥450 (n=78)** | **p-value** |
| --- | --- | --- | --- |
| **Demographics** |  |  |  |
| Age, years – median (IQR) | 72 (67–82) | 70 (67–82) | 0.08 |
| Male sex – n (%) | 28 (66.7) | 53 (67.9) | 0.89 |
| Comorbidities |  |  |  |
| - Chronic kidney disease – n (%) | 18 (42.9) | 22 (28.2) | 0.10 |
| - Diabetes mellitus – n (%) | 14 (33.3) | 19 (24.4) | 0.31 |
| - Chronic heart failure – n (%) | 20 (47.6) | 30 (38.5) | 0.33 |
| Severity of illness |  |  |  |
| - Critically ill – n (%) | 38 (90.5) | 48 (61.5) | <0.001 |
| - APACHE II score – median (IQR) | 18.6 (16–21) | 17.3 (16–21) | 0.01 |
| Complications |  |  |  |
| - Septic shock – n (%) | 9 (21.4) | 4 (5.1) | 0.01 |
| - Persistent bacteremia – n (%) | 12 (28.6) | 18 (23.1) | 0.52 |
| - New-onset/worsening heart failure – n (%) | 23 (54.8) | 33 (42.3) | 0.20 |
| Initial vancomycin regimen |  |  |  |
| - No loading dose – n (%) | 30 (71.4) | 28 (35.9) | <0.001 |
| - Initial dose <15 mg/kg – n (%) | 26 (61.9) | 18 (23.1) | <0.001 |
