# Supplementary material for: The optimal target of vancomycin area under the curve in early or later phase on clinical outcomes and nephrotoxicity in patients with enterococcal infective endocarditis: how much is enough?
Source: BMC Infect Dis. 2026 Mar 7;26:759. doi: 10.1186/s12879-026-12993-5 (PMC13081359; doi:10.1186/s12879-026-12993-5)
Supplement: Supplementary file 2 — Supplementary Material 2 [file 12879_2026_12993_MOESM2_ESM.docx]

**Supplementary Table 2. Baseline characteristics stratified by early vancomycin exposure (AUCss/MIC <420 vs ≥420)**

| **Variable** | **AUCss/MIC <420 (n=28)** | **AUCss/MIC ≥420 (n=92)** | **p-value** |
| --- | --- | --- | --- |
| **Demographics** |  |  |  |
| Age, years – median (IQR) | 72 (68–82) | 71 (67–82) | 0.15 |
| Male sex – n (%) | 17 (60.7) | 64 (69.6) | 0.38 |
| **Comorbidities** |  |  |  |
| - Chronic kidney disease – n (%) | 9 (32.1) | 10 (10.9) | 0.02 |
| - Diabetes mellitus – n (%) | 9 (32.1) | 24 (26.1) | 0.51 |
| - Chronic heart failure – n (%) | 14 (50.0) | 36 (39.1) | 0.29 |
| Severity of illness |  |  |  |
| - Critically ill – n (%) | 24 (85.7) | 62 (67.4) | 0.08 |
| - APACHE II score – median (IQR) | 20.1 (15–21) | 18.4 (16–21) | 0.010 |
| Complications |  |  |  |
| - Septic shock – n (%) | 5 (17.9) | 8 (8.7) | 0.14 |
| - Persistent bacteremia – n (%) | 9 (32.1) | 21 (22.8) | 0.30 |
| - New-onset/worsening heart failure – n (%) | 15 (53.6) | 41 (44.6) | 0.40 |
| **Management** |  |  |  |
| - Cardiac surgery – n (%) | 16 (57.1) | 68 (73.9) | 0.07 |
| **Concomitant nephrotoxic drugs** |  |  |  |
| - Gentamicin – n (%) | 24 (85.7) | 61 (66.3) | 0.05 |
| - Furosemide – n (%) | 13 (46.4) | 33 (35.9) | 0.29 |
| - Colistin – n (%) | 3 (10.7) | 4 (4.3) | 0.19 |
| - ≥2 nephrotoxic drugs – n (%) | 15 (53.6) | 26 (28.3) | 0.022 |
